# Impact of left ventricular ejection fraction on the effect of beta-blocker therapy on 1-year mortality in acute coronary syndrome patients

**DOI:** 10.1093/ehjcvp/pvaf062

**Published:** 2025-08-12

**Authors:** Micha T Maeder, Fabienne Foster-Witassek, Dragana Radovanovic, Marco Roffi, Giovanni Pedrazzini, Hans Rickli

**Affiliations:** Cardiology Department, HOCH Health Ostschweiz, Kantonsspital St. Gallen, Rorschacherstrasse 95, 9007 St. Gallen, Switzerland; AMIS Plus Data Center, Epidemiology, Biostatistics and Prevention Institute, University of Zurich, 8001 Zurich, Switzerland; AMIS Plus Data Center, Epidemiology, Biostatistics and Prevention Institute, University of Zurich, 8001 Zurich, Switzerland; Department of Cardiology, Geneva University Hospitals, 1205 Geneva, Switzerland; Department of Cardiology, Cardiocentro Ticino, 6900 Lugano, Switzerland; Cardiology Department, HOCH Health Ostschweiz, Kantonsspital St. Gallen, Rorschacherstrasse 95, 9007 St. Gallen, Switzerland

**Keywords:** Acute coronary syndrome, Beta-blocker, Left ventricular ejection fraction, Mortality

## Abstract

**Aims:**

While the beneficial effect of beta-blocker (BB) therapy for acute coronary syndrome (ACS) patients with a left ventricular ejection fraction (LVEF) of 40% is established, its role in those with LVEF > 40% is controversial. We assessed the relationship between BB therapy at discharge and 1-year mortality according to LVEF in a large contemporary acute coronary syndrome (ACS) cohort.

**Methods and results:**

Patients enrolled in the Acute Myocardial Infarction in Switzerland (AMIS) Plus registry between 2005 and 2024 with information on BB at discharge, LVEF, and 1-year mortality were studied. The association between BB therapy and 1-year mortality and the interaction with LVEF (>40% vs. ≤40%) were analysed. Among 7820 patients (65% with ST-segment elevation myocardial infarction), 1570 (20.1%) had LVEF ≤ 40%. At discharge, 6211/7820 (79.4%) patients were on BB (LVEF > 40%, 78.1%; LVEF ≤ 40%, 84.5%). One-year mortality was higher in patients with LVEF ≤ 40% vs. >40% (7.1% vs. 2.3%; *P* < 0.001). Overall, BB therapy was associated with reduced mortality [unadjusted odds ratio 0.67 (95% confidence interval 0.51–0.89); *P* = 0.005]. Among patients with LVEF ≤ 40%, mortality was lower in patients with BB compared with those without (5.9% vs. 14%; *P* < 0.001). In contrast, in patients with LVEF > 40%, mortality did not differ between patients with and without BB (2.1% vs. 2.6%; *P* = 0.3). A statistically significant interaction between BB therapy and LVEF stratum was identified (*p*_interaction_ = 0.02).

**Conclusion:**

Data from our large, nationwide registry suggest an overall benefit of BB therapy at discharge on 1-year mortality in ACS with most of the survival advantage observed in patients with LVEF < 40%.

## Graphical Abstract

**Graphical Abstract:**
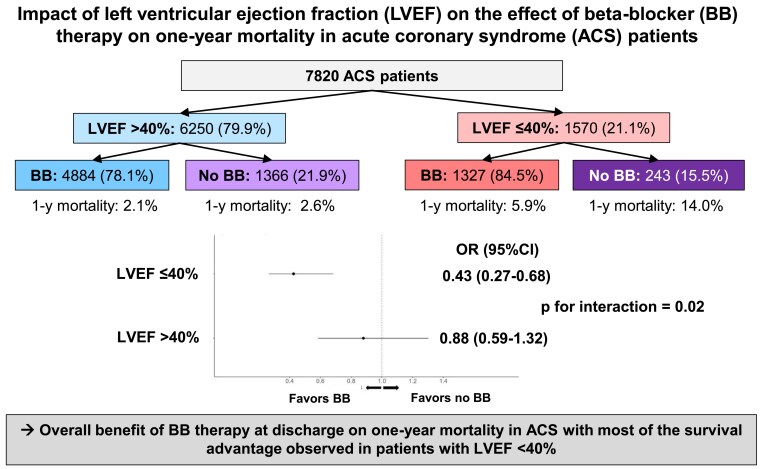
OR, odds ratio; PCI, percutaneous coronary intervention; 95% CI, 95% confidence interval.

## Introduction

In clinical practice, patients with acute coronary syndromes (ACSs) are commonly treated with beta-blockers (BBs). The underlying rationale includes the reduction of myocardial oxygen consumption by reduction of heart rate and contractility, the prevention of arrhythmias, and the attenuation of an unfavourable left ventricular remodelling.^1,2^ These mechanisms were particularly relevant in the pre-reperfusion era, a time in which most studies supporting the use of BB were performed.^2,3^ However, BB treatment can be associated with side effects impacting both quality of life and adherence to therapy.^4^ Although routine BB therapy after ACS is still recommended by guidelines,^5,6^ this strategy has come under scrutiny because data providing a solid rationale for such a practice are only available for patients with left ventricular ejection fraction (LVEF) < 40% and/or heart failure (HF).^1,2,7^ In contrast, in the era of coronary reperfusion and contemporary medical therapy, data on the efficacy of BB therapy in ACS patients with mildly reduced or preserved LV systolic function have been limited and conflicting.^1,2,8,9^ Recently, two adequately powered trials addressing the issue of contemporary post-ACS BB treatment were published, the *Randomized Evaluation of Decreased Usage of Beta-Blockers after Acute Myocardial Infarction* (REDUCE-AMI) and the *Assessment of Beta-blocker interruption 1 Year after an uncomplicated myocardial Infarction on Safety and Symptomatic cardiac events requiring hospitalization* (ABYSS).^10,11^ The REDUCE-AMI trial comparing a strategy of BB with no BB therapy initiated within a few days after ACS in patients undergoing revascularization and with LVEF ≥ 50% found no significant difference with regard to the primary composite endpoint of all-cause mortality or non-fatal ACS.^10^ On the other hand, the ABYSS trial evaluating the safety of the interruption vs. continuation of established BB therapy in patients in the chronic phase after an ACS with LVEF ≥ 40% at the time of randomization could not prove the non-inferiority of the BB interruption strategy.^11^ Thus, these two trials had a different design and setting and revealed at least in part conflicting results. Additional registry data are therefore needed to better understand which post-ACS patients may benefit from BB therapy in an unselected patient population. We report data on the association between BB therapy at hospital discharge and 1-year mortality stratified by LVEF (≤40% vs. >40%) from a nationwide ACS registry.

## Methods

### Patients

This is an analysis of patients enrolled in the Acute Myocardial Infarction in Switzerland (AMIS) Plus registry (www.amis-plus.ch), whose details have been reported previously. In brief, AMIS Plus is a large, prospective national registry in Switzerland that has been collecting hospital data on patients with ACS using a standardized questionnaire since 1997. Since its foundation, 84 centres have contributed data, and 63 centres have contributed data to this analysis. Collected data include cardiovascular risk factors, comorbidities, clinical presentation, and LVEF, as well as patient management. In the standardized questionnaire, LVEF was reported as follows: <30%, 30–40%, and >40%. Since 2006, a proportion of patients who consented at the time of the index hospitalization were also contacted by phone interview at 1 year by trained AMIS Plus data centre staff for follow-up. This study is in accordance with the Declaration of Helsinki regarding investigations on humans and was approved by the Swiss National Ethical Committee for Clinical Studies, the Board for Data Security, and all cantonal ethic committees (NCT 01,305,785).

For the present analysis, we included patients discharged after an ACS with (i) information on BB therapy at hospital discharge, (ii) information on LVEF by echocardiography, and (iii) information on 1-year follow-up.

### Statistical analysis

Continuous data are presented as median (interquartile range), and categorical data are given as numbers and percentages. Baseline characteristics of patients with and without BB therapy were compared using Wilcoxon rank-sum tests (i.e. Mann–Whitney *U*-tests) and χ^2^ tests or Fisher’s exact tests as appropriate. The primary endpoint of the analysis was mortality at 1 year. Multivariable logistic regression, with the co-variables age, sex, ACS type, previous ACS, LVEF (≤40% vs. >40%), HF, hypertension, diabetes, and percutaneous coronary intervention (PCI) at index event, was performed to analyse the adjusted association of BB treatment and 1-year mortality. To test the hypothesis that BBs have a stronger impact on mortality in patients with impaired LVEF, the same model was repeated including additionally an interaction term between BB at discharge and LVEF (≤40% vs. >40%). A *P* value of <0.05 was considered statistically significant. All analyses were performed using R, version 4.3.1.

## Results

**Figure 1:**
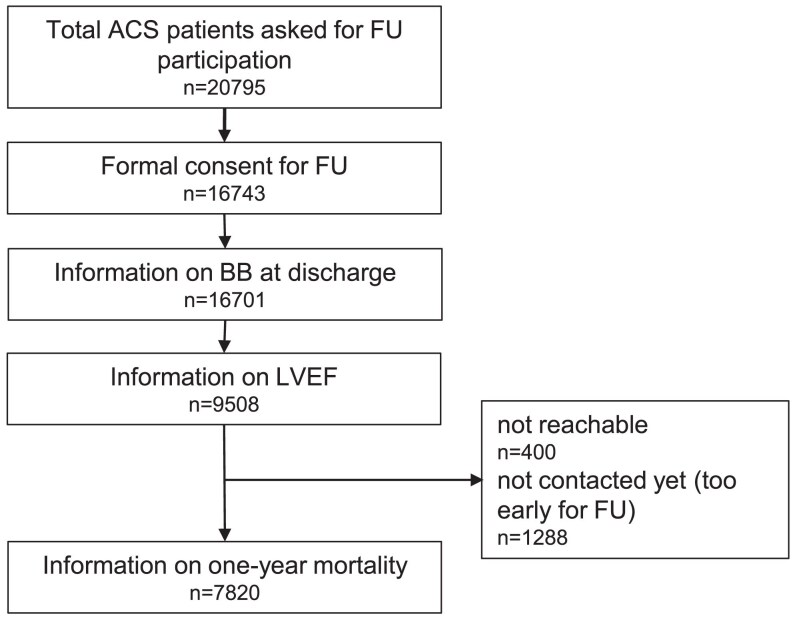
Flow chart showing the selection of the final study population.

Between 2005 und 2024, 20 795 ACS patients were included into the registry and asked for agreement for a 1-year follow-up. Among those, 7820 patients provided consent; had complete information on BB status at discharge, on LVEF at the index hospitalization, and on one-year follow-up; and were included in the present analysis (*Figure 1*). There were some differences between included and excluded patients (see Supplementary material online, *Table S1*). The proportion of patients with ST-segment elevation myocardial infarction (STEMI; 65% vs. 57%) and Killip class >II (4.5% vs. 3.7%) was higher in the included patients, there were minor differences in the risk factor profile, and the use of PCI was slightly more common in the included patient group (88% vs. 87%) (see Supplementary material online, *Table S1*).

In the present study population, the proportions of patients with STEMI, non-ST-segment elevation myocardial infarction (NSTEMI), and unstable angina were 65%, 33%, and 2%. In 6250/7820 (79.9%) patients, LVEF was >40%, whereas in 1570/7820 (20.1%), LVEF was ≤40%. At hospital discharge, 6211/7820 (79.4%) patients were on BB therapy (LVEF > 40%, 78.1%; LVEF ≤ 40%, 84.5%).

### Baseline characteristics and treatment for ACS

In the Supplementary material online, *Table S2*, characteristics of patients with vs. without BB are compared in the entire population. Patients on BB were younger; were more likely to have hypertension, diabetes, obesity, a history of a previous ACS, and HF; were more likely to have presented with STEMI and with out-of-hospital resuscitation; were less likely to have chronic lung disease; and had higher admission diastolic blood pressure and heart rate but lower LVEF (higher proportion of patients with LVEF ≤ 40%) than those not on BB.

In *Table 1*, clinical characteristics and treatments for the index ACS are compared between patients with and without BB therapy at hospital discharge, stratified for LVEF (≤40% vs. >40%). There were minor differences in medical history and risk factor profile between patients with and without BB therapy. Important differences included a higher proportion of patients undergoing PCI in the BB group in both LVEF strata, a higher proportion of patients with STEMI and out-of-hospital cardiac arrest in the BB group in patients with LVEF > 40%, and a higher proportion of patients treated with aspirin and angiotensin-converting enzyme inhibitor therapy in the BB group in patients with LVEF ≤ 40%.

**Table 1:** Patient characteristics and 1-year mortality in acute coronary syndrome (ACS) patients with vs. without beta-blocker (BB) at discharge stratified according to left ventricular ejection fraction (LVEF) > 40% vs. ≤40%

Characteristic	LVEF > 40%					LVEF ≤ 40%				
	*n*	Overall (*n* = 6250)	No BB (*n* = 1366)	BB (*n* = 4884)	*P* value	*n*	Overall (*n* = 1570)	No BB (*n* = 243)	BB (*n* = 1327)	*P* value
Age (years)	6250	64.8 (55.5–74.4)	65.6 (55.8–75.1)	64.7 (55.4–74.2)	0.074	1570	69.1 (59.3–77.7)	71.8 (63.0–80.3)	68.6 (58.7–77.2)	**<0**.**001**
Sex	6250				0.6	1570				0.11
Male		4651 (74%)	1024 (75%)	3627 (74%)			1194 (76%)	175 (72%)	1019 (77%)	
Female		1599 (26%)	342 (25%)	1257 (26%)			376 (24%)	68 (28%)	308 (23%)	
ACS type	6250				**<0**.**001**	1570				0.4
STEMI		3910 (63%)	756 (55%)	3154 (65%)			1150 (73%)	178 (73%)	972 (73%)	
NSTEMI		2167 (35%)	564 (41%)	1603 (33%)			399 (25%)	64 (26%)	335 (25%)	
Unstable angina		173 (2.8%)	46 (3.4%)	127 (2.6%)			21 (1.3%)	1 (0.4%)	20 (1.5%)	
Killip class	6241				0.8	1567				0.3
Class I		5711 (92%)	1255 (92%)	4456 (91%)			1109 (71%)	160 (66%)	949 (72%)	
Class II		355 (5.7%)	77 (5.6%)	278 (5.7%)			282 (18%)	51 (21%)	231 (17%)	
Class III		83 (1.3%)	16 (1.2%)	67 (1.4%)			90 (5.7%)	17 (7.0%)	73 (5.5%)	
Class IV		92 (1.5%)	17 (1.2%)	75 (1.5%)			86 (5.5%)	15 (6.2%)	71 (5.4%)	
Killip class >II	6241	175 (2.8%)	33 (2.4%)	142 (2.9%)	0.3	1567	176 (11%)	32 (13%)	144 (11%)	0.3
Pre-hospital resuscitation	6250	246 (3.9%)	34 (2.5%)	212 (4.3%)	**0**.**002**	1570	106 (6.8%)	15 (6.2%)	91 (6.9%)	0.7
Systolic blood pressure (mmHg)	6087	136 (119–155)	137 (119–155)	136 (119–155)	0.9	1536	130 (112–150)	128 (110–147)	130 (112–150)	0.059
Diastolic blood pressure (mmHg)	6084	80 (70–90)	80 (69–90)	80 (70–90)	**0**.**011**	1534	80 (70–90)	79 (68–89)	80 (70–90)	**0**.**019**
Heart rate (b.p.m.)	6090	75 (65, 87)	72 (60–84)	76 (66–88)	**<0**.**001**	1532	83 (71–99)	80 (68–100)	84 (72–99)	0.087
LVEF	6250				>0.9	1570				>0.9
<30%		0 (0%)	0 (0%)	0 (0%)			348 (22%)	54 (22%)	294 (22%)	
30–40%		0 (0%)	0 (0%)	0 (0%)			1222 (78%)	189 (78%)	1033 (78%)	
>40%		6250 (100%)	1366 (100%)	4884 (100%)			0 (0%)	0 (0%)	0 (0%)	
**Comorbidities and risk factors**										
Past history of ACS	6181	690 (11%)	137 (10%)	553 (11%)	0.2	1548	339 (22%)	41 (17%)	298 (23%)	**0**.**050**
History of heart failure	6179	80 (1.3%)	14 (1.0%)	66 (1.4%)	0.3	1546	98 (6.3%)	12 (5.0%)	86 (6.6%)	0.4
Peripheral vascular disease	6179	236 (3.8%)	62 (4.6%)	174 (3.6%)	0.10	1546	109 (7.1%)	23 (9.6%)	86 (6.6%)	0.10
Cerebrovascular disease	6179	241 (3.9%)	50 (3.7%)	191 (4.0%)	0.6	1546	113 (7.3%)	24 (10%)	89 (6.8%)	0.081
Hemiplegia	6179	22 (0.4%)	7 (0.5%)	15 (0.3%)	0.3	1546	11 (0.7%)	2 (0.8%)	9 (0.7%)	0.7
Dementia	6179	18 (0.3%)	5 (0.4%)	13 (0.3%)	0.6	1546	25 (1.6%)	4 (1.7%)	21 (1.6%)	>0.9
Chronic lung disease	6179	307 (5.0%)	82 (6.0%)	225 (4.7%)	**0**.**039**	1546	124 (8.0%)	35 (15%)	89 (6.8%)	**<0**.**001**
Peptic ulcer disease	6179	83 (1.3%)	12 (0.9%)	71 (1.5%)	0.10	1546	29 (1.9%)	8 (3.3%)	21 (1.6%)	0.11
Moderate to severe renal disease	6179	314 (5.1%)	65 (4.8%)	249 (5.2%)	0.6	1546	158 (10%)	32 (13%)	126 (9.6%)	0.083
Cancer	6179	316 (5.1%)	75 (5.5%)	241 (5.0%)	0.4	1546	96 (6.2%)	25 (10%)	71 (5.4%)	**0**.**003**
CCI > 1	6179	1036 (17%)	233 (17%)	803 (17%)	0.7	1546	459 (30%)	84 (35%)	375 (29%)	0.050
Current smoker	5795	2047 (35%)	444 (36%)	1603 (35%)	0.8	1440	480 (33%)	76 (35%)	404 (33%)	0.7
Hypertension	6043	3680 (61%)	777 (58%)	2903 (62%)	**0**.**025**	1506	984 (65%)	132 (57%)	852 (67%)	**0**.**006**
Dyslipidaemia	5689	3843 (68%)	898 (70%)	2945 (67%)	**0**.**011**	1410	926 (66%)	125 (59%)	801 (67%)	**0**.**020**
Diabetes mellitus	6066	1023 (17%)	202 (15%)	821 (17%)	0.063	1520	370 (24%)	54 (23%)	316 (25%)	0.6
Obesity (BMI > 30 kg/m^2^)	5937	1230 (21%)	210 (16%)	1020 (22%)	**<0**.**001**	1470	307 (21%)	31 (14%)	276 (22%)	**0**.**005**
**Regular medication before admission**										
Aspirin	5779	1490 (26%)	321 (26%)	1169 (26%)	0.9	1462	520 (36%)	68 (30%)	452 (37%)	**0**.**049**
Beta-blocker	5753	1291 (22%)	114 (9.2%)	1177 (26%)	**<0**.**001**	1450	409 (28%)	30 (13%)	379 (31%)	**<0**.**001**
Angiotensin-converting inhibitor	5725	935 (16%)	187 (15%)	748 (17%)	0.2	1442	307 (21%)	31 (14%)	276 (23%)	**0**.**003**
Calcium channel blocker	5712	759 (13%)	155 (13%)	604 (13%)	0.4	1439	202 (14%)	35 (16%)	167 (14%)	0.5
Angiotensin receptor blocker	5734	1014 (18%)	240 (19%)	774 (17%)	0.082	1436	259 (18%)	47 (21%)	212 (17%)	0.2
**Treatment during admission**										
Aspirin, immediate treatment	6239	6059 (97%)	1319 (97%)	4740 (97%)	0.3	1565	1493 (95%)	229 (94%)	1264 (96%)	0.3
PCI	6144	5492 (89%)	1168 (87%)	4324 (90%)	**0**.**001**	1533	1278 (83%)	182 (76%)	1096 (85%)	**0**.**001**

Data are reported as number (percentages) or median (interquartile range).

The numbers in bold are statistically significant.

BMI, body mass index; CCI, Charlson comorbidity index; NSTEMI, non-ST-segment elevation myocardial infarction; PCI, percutaneous coronary intervention; STEMI, ST-segment elevation myocardial infarction.

### One-year mortality according to BB therapy status and LVEF

**Figure 2:**
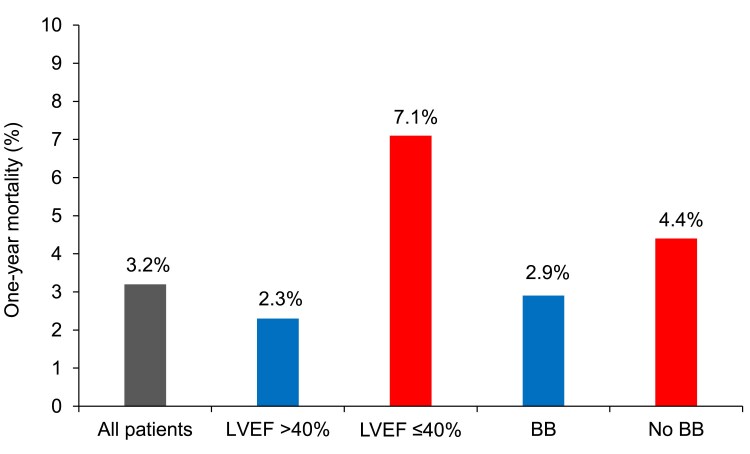
One-year mortality in the entire acute coronary syndrome population and in patients with vs. without beta-blocker at discharge and in patients with left ventricular ejection fraction (LVEF) ≤ 40% vs. >40%. BB, beta-blocker.

**Figure 3:**
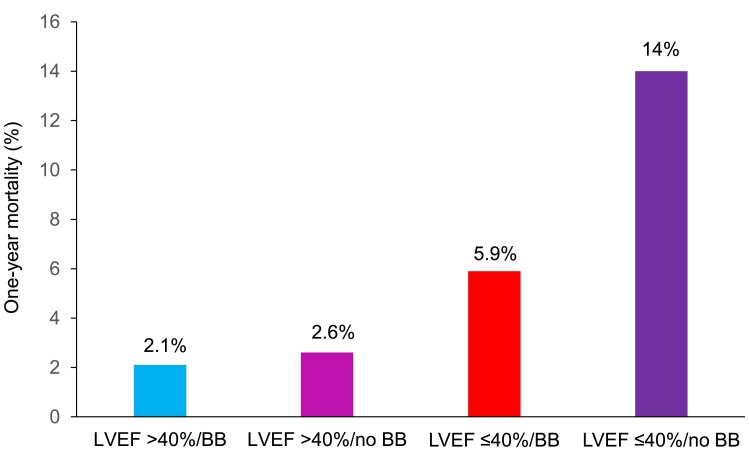
One-year mortality in patients with vs. without beta-blocker at discharge stratified by left ventricular ejection fraction ≤ 40% vs. >40%. BB, beta-blocker; LVEF, left ventricular ejection fraction.

In the entire population of patients discharged after ACS, there were 253/7820 (3.2%) deaths after 1 year (*Figure 2*). Crude mortality was higher in patients with LVEF ≤ 40% compared with those with LVEF > 40% (7.1% vs. 2.3%; *P* < 0.001; *Figure 2*). Overall, mortality was lower in patients discharged on BB compared with those without {2.9% vs. 4.4%; *P* = 0.005; *Figure 2*; unadjusted odds ratio (OR) 0.67 [95% confidence interval (CI) 0.51–0.89]; *P* = 0.005}. This was driven by a significantly lower mortality in patients on BB vs. those without BB [78/1327 (5.9%) vs. 34/243 (14%); *P* < 0.001] among patients with LVEF ≤ 40% (*Figure 3*). In contrast, mortality did not differ in patients with vs. without BB [105/4884 (2.1%) vs. 36/1366 (2.6%); *P* = 0.3] in patients with LVEF > 40% (*Figure 3*).

### Predictors of 1-year mortality

In the overall cohort, BB therapy at discharge was independently associated with a reduction in mortality at 1 year [adjusted OR 0.66 (95% CI 0.49–0.90); *P* = 0.007]. The predictors of one-year death according to the multivariable analysis are shown in *Table 2*. Patients with LVEF ≤ 40% had a more than two-fold risk of death compared with those with LVEF > 40% [adjusted OR 2.36 (95% CI 1.77–3.13); *P* < 0.001]. Protective factors other than BB and LVEF > 40% included younger age, absence of a prior ACS, HF, or diabetes, as well as treatment with PCI during the index hospitalization (*Table 2*).

**Table 2:** Predictors of 1-year mortality according to multivariable logistic regression in acute coronary syndrome patients

Predictors	One-year mortality		
	Odds ratio	95% confidence interval	*P* value
Age (per year)	1.08	1.07–1.10	**<0.001**
Female sex	0.73	0.53–1.00	0.051
Left ventricular ejection fraction ≤ 40%	2.36	1.77–3.13	**<0.001**
STEMI	1.00	(referent)	
NSTEMI	0.89	0.66–1.20	0.465
Unstable angina	0.81	0.30–1.79	0.630
Previous acute coronary syndrome	1.48	1.06–2.04	**0.019**
History of heart failure	1.99	1.19–3.22	**0.006**
Hypertension	0.78	0.58–1.07	0.125
Diabetes	1.54	1.13–2.08	**0.005**
Percutaneous coronary intervention	0.64	0.46–0.90	**0.008**
Beta-blocker at discharge	0.66	0.49–0.90	**0.007**

The numbers in bold are statistically significant.

NSTEMI, non-ST-segment elevation myocardial infarction; STEMI, ST-segment elevation myocardial infarction.

**Figure 4:**
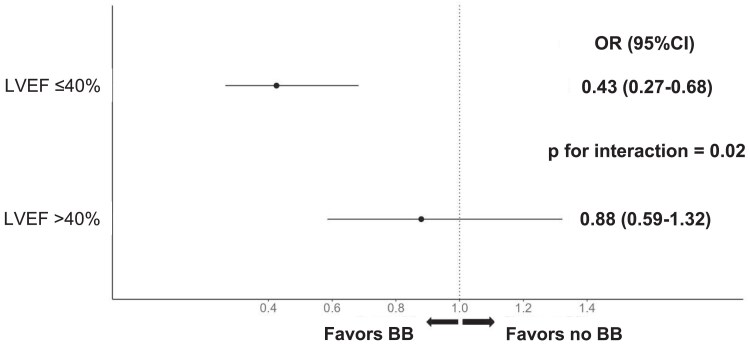
Probability of death according to beta-blocker therapy and left ventricular ejection fraction stratum. Odds ratios and 95% confidence intervals are reported. The *P* value refers to the interaction between the LVEF stratum and BB therapy. BB, beta-blocker; LVEF, left ventricular ejection fraction; ORs, odds ratios; 95% CIs, 95% confidence intervals.

In the multivariable analysis that included the interaction term, a significant interaction between the LVEF stratum and BB therapy status was found [BB at discharge × LVEF ≤ 40%: OR 0.48 (95% CI 0.26–0.90); *p*_interaction_ = 0.02], as illustrated in *Figure 4*, with a stronger benefit of BB on 1-year mortality in patients with LVEF ≤ 40%.

## Discussion

The present analysis from a large well-characterized, contemporary ACS population revealed a significant association between BB therapy at hospital discharge and lower 1-year mortality in the one-fifth of patients with LVEF ≤ 40%, which is fully in line with existing data and guideline recommendations for this subset of post-ACS patients.^5,6^ In the four-fifth of patients with LVEF > 40%, 1-year mortality was 2.4 times lower than in patients with LVEF ≤ 40%, and there was no impact of BB therapy on mortality. The significant interaction between the LVEF stratum and the effect of BB on mortality further supports the notion of a differential impact on mortality of BB according to LVEF, with a preferential benefit observed in patients with LVEF ≤ 40% (*Graphical abstract*).

The evidence regarding the role of BB therapy for the early post-ACS period (first year) in patients without significant LV dysfunction (i.e. LVEF > 40–50%) is limited to one randomized trial^10^ and several retrospective studies. Meta-analyses of these studies have revealed conflicting findings and suggested a publication bias.^8^ Many studies (some of them suggesting a benefit of BB therapy, e.g. Choo *et al.*^12^) had relevant limitations including their retrospective design,^12–14^ lack of information on LVEF,^13,14^ and lack of a systematically conducted follow-up.^14^ Some studies were based on insurance service data and coded diagnoses with inherent limitations.^14^ The randomized Carvedilol Post-Intervention Long-Term Administration in Large-scale Randomized Controlled Trial (CAPITAL-RCT) including 801 STEMI patients with LVEF ≥ 40% randomized to carvedilol vs. no carvedilol in an open-label design found no difference with regard to (i) the primary endpoint of all-cause death, ACS, and hospitalization for HF and (ii) any coronary revascularization after a median follow-up of 3.9 years (6.8% vs. 7.9% and 20.3% vs. 17.7%, respectively).^9^ However, the trial was underpowered. The recent REDUCE-AMI trial comparing a strategy of BB with no BB therapy in 5020 ACS patients undergoing revascularization with LVEF ≥ 50% found no significant difference with regard to the primary composite endpoint of all-cause mortality or non-fatal ACS after a median follow-up of 3.5 years [BB: 7.9% vs. no BB: 8.3%; hazard ratio 0.96 (95% confidence interval 0.79–1.16); *P* = 0.64].^10^ In that trial, patients were included within 1 week (median: 2 days) after admission and received metoprolol (median dose: 100 mg) or bisoprolol (median dose: 5 mg) vs. placebo in an open-label setting. This trial was the first adequately powered trial investigating the effect of BB therapy in ACS patients with preserved LVEF in a contemporary setting characterized by coronary reperfusion and optimal secondary prevention. Limitations included the open-label design, the lower-than-expected event rate, and a notable cross-over rate (18% of patients assigned to BB stopped it, and 14% of patients assigned to no BB were started on a BB).^1,15^

Differing from most previous analyses, the present study was prospective in nature. In addition, the group of interest, i.e. patients with LVEF > 40%, had a similar size (*n* > 5000) to the population enrolled in REDUCE-AMI trial.^10^ Mortality in the present study (LVEF > 40% stratum) was higher than in REDUCE-AMI^10^ (2.3% at 1 year vs. 4.0% after a median follow-up of 3.5 years), which may be explained by the different LVEF inclusion criteria (LVEF > 40% vs. LVEF ≥ 50%) and the fact that we studied a real-life cohort as opposed to a selected trial population. Our data expand the findings of the REDUCE-AMI trial^10^ to a broader real-life patient population, further questioning the practice of routine BB therapy in ACS patients with LVEF > 40%.^1,2,7^

As a limitation of the present analysis, we cannot explore the effects of BB therapy in patients with LVEF ≥ 50% vs. those with LVEF > 40% but <50% because LVEF data were collected as category (i.e. <30%, 30–40%, and >40%). The group of patients with LVEF between 40% and 50% post-MI was included in the recently published ABYSS trial.^11^ The ABYSS trial evaluated the safety and impact on quality of life of the interruption vs. continuation of established BB therapy in the chronic phase after an ACS (defined as occurring >6 months prior to randomization, while the median interval was 2.9 years) in patients with LVEF ≥ 40% at the time of randomization (the median LVEF being 60%; 23.4% with LVEF between 40% and 50%). The trial could not prove the non-inferiority of the BB interruption strategy because a numerically higher incidence of the primary composite endpoint of death, non-fatal ACS, non-fatal stroke, and hospitalization of cardiovascular reasons was observed in the interruption group. While the numbers of death, ACS, and stroke were similar in both groups, the difference was driven by cardiovascular hospitalization due to coronary-related reasons. In addition, no advantage in terms of quality of life was observed in the BB interruption group.^11^ This trial investigated a different setting than REDUCE-AMI, i.e. enrolled patients with LVEF ≥ 40% after receiving BB for nearly 3 years. Importantly, LVEF at the time of index ACS, coronary status, and specific reasons for BB prescription was unknown in ABYSS.^11^ Potential mechanisms explaining why the non-inferiority of BB discontinuation was not met may include worsening of LVEF, unmasking of previously silent ischaemia, and the occurrence of supraventricular and ventricular arrhythmias. Importantly, in the ABYSS trial, BB interruption led to a sustained increase in heart rate and systolic and diastolic blood pressure despite an increase in antihypertensive drugs,^16^ which could represent a plausible explanation for the occurrence of coronary events. Overall, the ABYSS trial^11^ results are not informative on whether or not BB should be administered in all patients with an LVEF > 40% after an ACS.

Two ongoing randomized BB trials including ACS patients with LVEF ≥ 40%^17,18^ should provide more evidence. The tREatment with Beta-blockers after myOcardial infarction withOut reduced ejection fracTion (REBOOT) trial^17^ plans enrolling 8468 patients with ACS managed invasively (i.e. coronary angiography followed by PCI, surgical revascularization, or medical management), LVEF > 40%, and no history of HF who will be randomized to BB (substance and dose at the discretion of the treating physician) vs. no BB at discharge. The primary endpoint is a composite of all-cause death, non-fatal ACS, or HF hospitalization over a median follow-up period of 2.75 years.^17^ The Danish Trial of Beta Blocker therapy after myocardial infarction without heart failure (DANBLOCK) and the Norwegian BEtablocker Treatment after Acute Myocardial Infarction in revascularized patients without reduced left ventricular ejection fraction (BETAMI) trial, which have similar design and due to lower than expected inclusion rates were combined for analysis,^18^ aim to recruit ≈5700 patients with a recent ACS (within 14 days), LVEF ≥ 40%, and no HF and to randomize them to BB (substance and dose at the discretion of the treating physician) vs. no BB. The primary endpoint is a composite of recurrent ACS, incident HF, coronary revascularization, ischaemic stroke, all-cause mortality, malignant ventricular arrhythmia, or resuscitated cardiac arrest.^18^

Current guidelines^5,6^ still provide a strong recommendation for BB therapy for ACS patients irrespective of LVEF. The 2023 ACS guidelines of the European Society of Cardiology, published before the release of the results of REDUCE-AMI^10^ and ABYSS,^11^ recommended BB for all MI patients with LVEF ≤ 40% (Class I recommendation) and stated that BB should be considered for all ACS patients regardless of LVEF (Class IIa recommendation).^6^ The 2025 ACS guidelines of the American College of Cardiology and the American Heart Association (i.e. published after the release of REDUCE-AMI^10^ and ABYSS^11^) recommend the early (<24 h) initiation of BB therapy in all ACS patients (Class I recommendation; duration of BB therapy not specified for patients with ‘preserved’ LVEF). The authors acknowledge the results of the REDUCE-AMI trial^10^ but state that the evidence is currently insufficient for a change in practice and point to ongoing trials.^5^

In clinical practice, it is indeed not straightforward to integrate the results from REDUCE-AMI^10^ as well as our own observations. Accordingly, in the acute phase of an ACS, it is often not obvious how LVEF will evolve in patients with an acute complete occlusion involving a large myocardial territory (typically STEMI patients). In addition, in patients with multivessel coronary disease, revascularization is completed only within few weeks (staged PCI). Some ACS patients may also need BB for the treatment of arrhythmias (e.g. ventricular and supraventricular tachycardias). Therefore, it appears premature to change the current practice of routine BB treatment in STEMI patients with LVEF > 40% during the index hospitalization and during the first weeks after the event.

The present study has the limitations of an observational study. First of all, BB therapy was not randomized, and unmeasured confounders still may have played a role, even though the population was well characterized and data were collected prospectively. In particular, BB type and dose as well as treatment adherence may have played a role. Second, the follow-up was limited to 1 year. However, this is commonly considered the period after an acute MI in which BBs are useful. Third, LVEF was measured by echocardiography in all patients, but only LVEF categories (i.e. <30%, 30–40%, and >40%) were entered in the database. Therefore, an analysis with LVEF as a continuous variable or separate analysis of the group of patients with LVEF between 40% and 50% was not possible. The latter is a relevant limitation given that the LVEF threshold in REDUCE-AMI was 50%. In addition, LVEF was assessed by many different operators using different methods, which is a reflection of daily practice but which also has to be taken into account. Fourth, the present analysis is a subgroup analysis of selected patients, and the main finding is based on an interaction analysis. Still, this is one of the largest populations evaluating the association between BB therapy, LVEF, and post-ACS mortality. Finally, information on the implantation of defibrillators was not systematically collected, and thus the contribution of this therapy to mortality remains unclear.

## Conclusions

The analysis of this nationwide ACS registry suggests a greater benefit of BB therapy at discharge on 1-year mortality in patients with LVEF < 40%. The indications to give routine BB in ACS patients with preserved LVEF are less clear, and ongoing trials may provide further information.

## Supplementary Material

pvaf062_Supplementary_Data

## Data Availability

Due to data protection regulations related to the different hospitals involved in this study, the authors do not have authorization to provide unrestricted data access. However, after approval of the AMIS Plus Steering Committee and subsequent negotiation of an individual AMIS Plus module contract with the AMIS Plus Steering Committee, analysis files can be handed over to other researchers. Requests must be submitted to Prof. Dr Hans Rickli, President of the AMIS Plus Steering Committee (hans.rickli@h-och.ch) and Dr Dragana Radovanovic (Head of Data Center AMIS Plus, dragana.radovanovic@uzh.ch).
