# Supplementary material for: Impact of left ventricular ejection fraction on the effect of beta-blocker therapy on 1-year mortality in acute coronary syndrome patients
Source: Eur Heart J Cardiovasc Pharmacother. 2025 Aug 12;11(7):590–9. doi: 10.1093/ehjcvp/pvaf062 (PMC12582655; doi:10.1093/ehjcvp/pvaf062)
Supplement: pvaf062_Supplementary_Data [file pvaf062_supplementary_data.zip › R1_EHJCVPT_BB_Table_S1_21.07.2025.docx]

Supplemental Table S1. Comparison of included and excluded patients

|  | | | **Beta Blocker analysis** | |  |
| --- | --- | --- | --- | --- | --- |
| **Characteristic** | **N** | **Overall**  (n=20795) | **Not included**  (n=12975) | **Included**  (n=7820) | **p-value** |
| Age, years | 20795 | 65 (56-75) | 65 (56-76) | 66 (56-75) | 0.3 |
| Sex | 20795 |  |  |  | >0.9 |
| Male |  | 15539 (75%) | 9694 (75%) | 5845 (75%) |  |
| Female |  | 5256 (25%) | 3281 (25%) | 1975 (25%) |  |
| Diagnosis | 20795 |  |  |  | **<0.001** |
| STEMI |  | 12,396 (60%) | 7,336 (56%) | 5,060 (65%) |  |
| NSTEMI |  | 7,588 (36%) | 5,022 (39%) | 2,566 (33%) |  |
| Unstable angina |  | 811 (4%) | 617 (5%) | 194 (2%) |  |
| Killip class>II | 20744 | 829 (4.0%) | 478 (3.7%) | 351 (4.5%) | **0.004** |
| Cardiac insufficiency | 20536 | 505 (2.5%) | 327 (2.6%) | 178 (2.3%) | 0.3 |
| Peripheral vascular disease | 20536 | 944 (4.6%) | 599 (4.7%) | 345 (4.5%) | 0.5 |
| Cerebrovascular disease | 20536 | 931 (4.5%) | 577 (4.5%) | 354 (4.6%) | 0.8 |
| Chronic lung disease | 20536 | 1071 (5.2%) | 640 (5.0%) | 431 (5.6%) | 0.068 |
| Peptic ulcer disease | 20536 | 309 (1.5%) | 197 (1.5%) | 112 (1.4%) | 0.6 |
| Moderate to severe renal disease | 20537 | 1199 (5.8%) | 727 (5.7%) | 472 (6.1%) | 0.2 |
| Cancer | 20536 | 1081 (5.3%) | 669 (5.2%) | 412 (5.3%) | 0.7 |
| CCI>1 | 20536 | 4,075 (20%) | 2,580 (20%) | 1,495 (19%) | 0.2 |
| Current smoker | 19047 | 6934 (36%) | 4407 (37%) | 2527 (35%) | **<0.001** |
| Hypertension | 19956 | 12607 (63%) | 7943 (64%) | 4664 (62%) | **0.001** |
| Dyslipidemia | 18806 | 12167 (65%) | 7398 (63%) | 4769 (67%) | **<0.001** |
| Diabetes mellitus | 20092 | 3838 (19%) | 2445 (20%) | 1393 (18%) | **0.038** |
| Obesity (BMI>30) | 19329 | 4109 (21%) | 2572 (22%) | 1537 (21%) | 0.2 |
| PCI | 20420 | 17835 (87%) | 11065 (87%) | 6770 (88%) | **0.005** |
| Data are reported as number (percentages) or median (interquartile range).  BMI: body mass index; CCI: Charlson co-morbidity index; NSTEMI: Non-ST-segment elevation myocardial infarction; PCI: percutaneous coronary intervention; STEMI: ST-segment elevation myocardial infarction. | | | | | |
|  | | | | | |
