# Supplementary material for: Impact of left ventricular ejection fraction on the effect of beta-blocker therapy on 1-year mortality in acute coronary syndrome patients
Source: Eur Heart J Cardiovasc Pharmacother. 2025 Aug 12;11(7):590–9. doi: 10.1093/ehjcvp/pvaf062 (PMC12582655; doi:10.1093/ehjcvp/pvaf062)
Supplement: pvaf062_Supplementary_Data [file pvaf062_supplementary_data.zip › R1_EHJCVPT_BB_Table_S2_21.07.2025.docx]

Supplemental Table S2. Patient characteristics in acute coronary syndrome (ACS) patients with versus without beta-blocker (BB) therapy at discharge

|  | | | **Beta-blocker at discharge** | |  |
| --- | --- | --- | --- | --- | --- |
| **Characteristic** | **n** | **Overall**  (n=7820) | **No BB**  (n=1609) | **BB**  (n=6211) | **P value** |
| Age (years) | 7820 | 66 (56-75) | 67 (57-76) | 65 (56-75) | **0.019** |
| Gender | 7820 |  |  |  | 0.8 |
| Male |  | 5845 (75%) | 1199 (75%) | 4646 (75%) |  |
| Female |  | 1975 (25%) | 410 (25%) | 1565 (25%) |  |
| ACS type | 7820 |  |  |  | **<0.001** |
| STEMI |  | 5060 (65%) | 934 (58%) | 4126 (67%) |  |
| NSTEMI |  | 2566 (33%) | 628 (39%) | 1938 (31%) |  |
| Unstable angina |  | 194 (2%) | 47 (3%) | 147 (2%) |  |
| Killip class | 7808 |  |  |  | 0.8 |
| Class I |  | 6,820 (87%) | 1,415 (88%) | 5,405 (87%) |  |
| Class II |  | 637 (8.2%) | 128 (8.0%) | 509 (8.2%) |  |
| Class III |  | 173 (2.2%) | 33 (2.1%) | 140 (2.3%) |  |
| Class IV |  | 178 (2.3%) | 32 (2.0%) | 146 (2.4%) |  |
| Killip class >II | 7808 | 351 (4.5%) | 65 (4.0%) | 286 (4.6%) | 0.3 |
| Pre-hospital resuscitation | 7820 | 352 (4.5%) | 49 (3.0%) | 303 (4.9%) | **0.002** |
| Systolic blood pressure (mmHg) | 7623 | 135 (117-154) | 135 (117-153) | 135 (117-154) | 0.8 |
| Diastolic blood pressure (mmHg | 7618 | 80 (70-90) | 80 (69-90) | 80 (70-90) | **0.001** |
| Heart rate (bpm) | 7622 | 77 (66-90) | 73 (62-85) | 78 (67-90) | **<0.001** |
| Left ventricular ejection fraction (LVEF) | 7820 |  |  |  | **<0.001** |
| <30% |  | 348 (4.5%) | 54 (3.4%) | 294 (4.7%) |  |
| 30-40% |  | 1222 (16%) | 189 (12%) | 1033 (17%) |  |
| >40% |  | 6250 (80%) | 1366 (85%) | 4884 (79%) |  |
| LVEF | 7820 |  |  |  | **<0.001** |
| >40% |  | 6250 (80%) | 1366 (85%) | 4884 (79%) |  |
| ≤40% |  | 1570 (20%) | 243 (15%) | 1327 (21%) |  |
| **Comorbidities and risk factors:** |  |  |  |  |  |
| Past history of ACS | 7729 | 1029 (13%) | 178 (11%) | 851 (14%) | **0.004** |
| History of heart failure | 7725 | 178 (2.3%) | 26 (1.6%) | 152 (2.5%) | **0.043** |
| Peripheral vascular disease | 7725 | 345 (4.5%) | 85 (5.3%) | 260 (4.2%) | 0.063 |
| Cerebrovascular disease | 7725 | 354 (4.6%) | 74 (4.6%) | 280 (4.6%) | >0.9 |
| Hemiplegia | 7725 | 33 (0.4%) | 9 (0.6%) | 24 (0.4%) | 0.3 |
| Dementia | 7725 | 43 (0.6%) | 9 (0.6%) | 34 (0.6%) | >0.9 |
| Chronic lung disease | 7725 | 431 (5.6%) | 117 (7.3%) | 314 (5.1%) | **<0.001** |
| Peptic ulcer disease | 7725 | 112 (1.4%) | 20 (1.3%) | 92 (1.5%) | 0.5 |
| Moderate to severe renal disease | 7725 | 472 (6.1%) | 97 (6.1%) | 375 (6.1%) | >0.9 |
| Cancer | 7725 | 412 (5.3%) | 100 (6.3%) | 312 (5.1%) | 0.064 |
| CCI>1 | 7725 | 1495 (19%) | 317 (20%) | 1178 (19%) | 0.6 |
| Current smoker | 7235 | 2527 (35%) | 520 (35%) | 2007 (35%) | 0.6 |
| Hypertension | 7,549 | 4664 (62%) | 909 (58%) | 3755 (63%) | **<0.001** |
| Dyslipidemia | 7,099 | 4769 (67%) | 1023 (69%) | 3746 (67%) | 0.14 |
| Diabetes mellitus | 7586 | 1393 (18%) | 256 (16%) | 1137 (19%) | **0.021** |
| Obesity (BMI>30 kg/m^2^) | 7407 | 1537 (21%) | 241 (16%) | 1296 (22%) | **<0.001** |
| **Regular medication before admission:** |  |  |  |  |  |
| Aspirin | 7241 | 2,010 (28%) | 389 (26%) | 1621 (28%) | 0.14 |
| Beta-blocker | 7203 | 1700 (24%) | 144 (9.8%) | 1556 (27%) | **<0.001** |
| Angiotensin converting enzyme inhibitor | 7167 | 1242 (17%) | 218 (15%) | 1024 (18%) | **0.005** |
| Calcium channel blocker | 7151 | 961 (13%) | 190 (13%) | 771 (14%) | 0.6 |
| Angiotensin receptor blocker | 7170 | 1,273 (18%) | 287 (20%) | 986 (17%) | **0.037** |
| **Immediate treatment:** |  |  |  |  |  |
| Aspirin, immediate treatment | 7804 | 7552 (97%) | 1548 (96%) | 6004 (97%) | 0.3 |
| Percutaneous coronary intervention | 7677 | 6770 (88%) | 1350 (85%) | 5420 (89%) | **<0.001** |
| Data are reported as number (percentages) or median (interquartile range).  BMI: body mass index; CCI: Charlson co-morbidity index; NSTEMI: Non-ST-segment elevation myocardial infarction; STEMI: ST-segment elevation myocardial infarction. | | | | | |
|  | | | | | |
