# Supplementary material for: Impact of left ventricular ejection fraction on the effect of beta-blocker therapy on 1-year mortality in acute coronary syndrome patients
Source: Eur Heart J Cardiovasc Pharmacother. 2025 Aug 12;11(7):590–9. doi: 10.1093/ehjcvp/pvaf062 (PMC12582655; doi:10.1093/ehjcvp/pvaf062)
Supplement: pvaf062_Supplementary_Data [file pvaf062_supplementary_data.zip › R1_EHJCVP_Supplementum_acknowledgement.docx]

**Acknowledgement**

The authors would like to express their gratitude to the teams of the following hospitals (listed in alphabetical order with the names of the local principal investigators): Aarau, Kantonsspital (P Lessing); Affoltern am Albis, Spital (F Hess); Altdorf, Kantonsspital Uri (R Simon/S Gisler); Baden, Kantonsspital (U Hufschmid); Basel, St. Claraspital (L Altwegg); Basel, Universitätsspital (R Jeger); Bern, Beau-Site Klinik (S Trummler); Bern, Inselspital (S Windecker); Bern, Tiefenauspital (P Loretan); Biel, Spitalzentrum (C Roethlisberger); Bülach, Spital (G Mang); Burgdorf, Regionalspital Emmental (D Ryser); Davos, Spital (W Kistler); Einsiedeln, Regionalspital (S Stäuble); Flawil, Spital (G Freiwald); Fribourg, Hôpital cantonal (JC Stauffer/S Cook); Frutigen, Spital (K Bietenhard); Genève, Hôpitaux universitaires (M Roffi); Grenchen, Spital (R Schönenberger); Herisau, Kantonales Spital (M Bötschi); Horgen, See Spital (B Federspiel); Interlaken, Spital (EM Weiss); Kreuzlingen, Herzzentrum Bodensee (K Weber); La Chaux-de-Fonds, Hôpital (H Zender); Lachen, Regionalspital (I Poepping); Langnau im Emmental, Regionalspital (A Hugi); Laufenburg, Gesundheitszentrum Fricktal (E Koltai); Lausanne, Centre hospitalier universitaire vaudois (JF Iglesias/S Fournier); Lugano, Cardiocentro Ticino (G Pedrazzini); Luzern, Luzerner Kantonsspital (P Erne/F Cuculi); Männedorf, Kreisspital (T Heimes); Mendrisio, Ospedale regionale (A Pagnamenta); Meyrin, Hôpital de la Tour (P Urban/A Fassa); Moutier, Hôpital du Jura bernois (C Stettler); Münsingen, Spital (F Repond); Münsterlingen, Kantonsspital (F Widmer); Muri, Kreisspital für das Freiamt (C Heimgartner); Nyon, Group. Hosp. Ouest lémanique (R Polikar); Olten, Kantonsspital (S Bassetti/ S Ernst); Rheinfelden, Gesundheitszentrum Fricktal (HU Iselin); Rorschach, Spital (M Giger); Sarnen, Kantonsspital Obwalden (T Kaeslin); Schaffhausen, Kantonsspital (A Fischer); Schlieren, Spital Limmattal (T Herren); Scuol, Ospidal d'Engiadina Bassa (C Neumeier/G Flury); Sion, Hôpital du Valais (G Girod); Solothurn, Bürgerspital (R Vogel); Stans, Kantonsspital Nidwalden (B Niggli); St. Gallen, Kantonsspital (H Rickli); Sursee, Luzerner Kantonsspital (J Nossen); Thun, Spital (U Stoller); Uster, Spital (E Bächli/J Debrunner); Wetzikon, GZO Spital (U Eriksson); Winterthur, Kantonsspital (T Fischer); Wolhusen, Luzerner Kantonsspital (M Peter/Y Suter); Zofingen, Spital (S Gasser); Zollikerberg, Spital (R Fatio); Zürich, Hirslanden Klinik (C Wyss); Zürich, Hirslanden Klinik im Park (O Bertel); Zürich, Universitätsspital, Intensivmedizin (M Maggiorini); Zürich, Universitätsspital, Kardiologie (B Stähli); Zürich, Stadtspital Triemli (F Eberli); Zürich, Stadtspital Waid (S Christen).
